# On the Various Forms of Vulvitis

**Published:** 1860-05

**Authors:** 

**Affiliations:** Professor of Miewifery in the University of Edinburgh


### ON THE VARIOUS FORMS OF VULVITIS.

BY DR. SIMPSON, PROFESSOR OF MIEWIFERY IN THE UNIVERSITY OF EDINBURGH.

The vulva is liable to other types of inflammation besides the simple inflammatory or phlegmonous, and also quite independently of any syphilitic origin. Various forms of eruptive inflammation are liable to appear in this part of the body, and particularly in some females at the menstrual period, and in others in consequence of the acridity of the discharges which may pass from the vagina at other times, and under states of internal disease. Thus, these parts are the occasional seat of attacks of erythema, eczema, &c. Herpes occasionally appears, generally after some constitutional disorder, on the external labia or vulva, of the same form and running the same course as herpes labialis, or rather, as herpes preputialis in the male. Dr. Simpson has seen also very distinct herpes upon the cervix uteri. Various other forms of acute and inflammatory eruption may appear in these parts, but they require no special notice, as they require no treatment specially different from the same diseases when situated elsewhere. One of the most common varieties of eruption on the vulva consists of an affection like acne, *i. e.*, it consists of scattered tubercles, sometimes of considerable size, formed by inflammation of the follicles of the vulva and nymphæ. But there are some forms of specific inflammation, if we may so call them, of the vulva, some varieties of vulvitis, if we may so speak, which call more particularly for notice, such as palpillary vulvitis, purulent vulvitis, (which is liable to occur in infants) gangrenous vulvitis, or noma, &c.

1. *Papillary Vulvitis.*—This is a form of chronic inflammatory disease of the vulva which is fortunately not very frequently met with in practice, but when it does occur, it is usually accompanied with much distress, and is often very obstinate in the way of cure. By this term, papillary vulvitis, I mean a chronic inflammatory affection of the mucous membrane of the vulva, and sometimes of the vagina, in which that membrane presents an intensly red color, either in raised red patches on the edge of the lower vaginal folds, on the tips of the carunculæ mytiformes, or the nymphæ, or on the sides of the vulva, or more generally diffused over a large portion of the surface of the vulva. On these red patches or surfaces you find, on examination, the enlarged mucous papillæ standing out like the swollen villi of a raw and irritable tongue. Often some of the most elongated papillæ bleed at their tip when touched. Any rough pressure of the vulva gives pain to the patient. The disease may be found in the unmarried, but not frequently; it comes on after marriage, and produces such tenderness of the parts that sexual connection is attended with much suffering, or becomes impossible. Usually, indeed, there is a sort of spasmodic contraction of the orifice of the vagina aggravating the evil. Along with the red and enlarged papillæ you will sometimes, but not always, find intersperced the small follicular glands of the vulva, swollen and inflamed, and sometimes ulcerated.

*Treatment.*—Patients affected with this disease anxiously demand professional relief, as their happiness as well as their health is sometimes destroyed by it, and the treatment, as I have already hinted, is sometimes by no means easy. I have known the diseased surface treated heroically and perseveringly with a repetition of strong caustics; but the simplest and surest mode of cure, according to my experience, consists in the continued use of local astringent measures, combined when necessary with sedatives, such as a strong solution or liniment of tannin frequently applied during the day, and with morphia or other sedatives added to it. Or you may dissolve your astringent in glycerine, and in this form it will remain more permanently in contact with the diseased surface. Latterly, I have trusted principally to the daily free application of a saturated solution of perchloride of iron in glycerine—an application producing little, or indeed no pain —and, I think, with the happiest effects. General tonics. warm bathing, &c., must not at the same time be forgotten,

2. *Purulent Vulvitis, or Leucorrhœa in Infants.*—This is a form of inflammation of the vulva which is by no means infrequent in female infants and children. It is, perhaps, however, more interesting and important in its medical jurisprudence relations than in its purely practical aspect; for, very often, especially when it occurs in patients of the lower orders, the disease is properly imagined, by the relatives and friends of those attacked, to be the result of veneral infection The child is brought to a medical practitioner, under the idea that he will confirm this unhappy and dangerous notion. An excitable mother will, by threats and by suggestive and leading questions, get the frightened child to own some absurd and groundless tale, in confirmation of her maternal theory of the origin of the malady; and no doubt men have been repeatedly tried, and convicted, too, of imparting this disease to young children, by forced sexual connection, when they were totally innocent of such a crime, and when the affection had totally a different origin; for the purulent vulvitis, or leucorrhœa, seen in young female children, has, as a general rule, no more an impure sexual origin than has the inflammation of the eyes, throat or lungs. Like most other local inflammations, it usually arises first from some degree of deranged or impaired generel health in the patient, and secondly from the patient, when in this, it may be quite temporarily, depressed condition, being exposed to cold and wet, or the other exciting causes of inflammation. Want of cleanliness, the irritation of acid urine, &c., sometimes act as a local exciting cause. Like angina, diphtherite, and some forms of catarrh and dysentery, this inflammation of the mucous membrane of the vulva, like those inflammations which I have named of the mucous, pulmonary, and intestinal membrane, attacks more than one child in the same family, and may even prove partially endemic or epidemic in a district.

*Symptoms and Course.*—The disease usually begins with a local heat and itching; some degree of redness and swelling; pain and scalding in passing water, and sometimes uneasiness in walking; but for the first twelve or twenty-four hours, there is no discharge from the mucous membrane.—But you will seldom see the disease in this early stage. Almost always you are called upon to see or prescribe for the patient, because the affection has so far run on in its course, that there is already more or less discharge from the vulva. The discharge is, at first, of a thin mucous character, but rapidly becomes purulent and yellow, or yellowish-green in its tint. Occasionally the discharge becomes extremely profuse. It is apt to become thick and hardened along the outer edges of the external labia, binding these opposed edges slightly together by a kind of imperfect crescentic crust; but when the labiæ are drawn asunder, the whole surface of the vulva is found to be covered over by a layer of the liquid puriform secretion. The disease very rarely extends upwards into either the vaginal or urethral canals. Occasionally there is, at points, an appearance of vesicular or pustular eruption; and, indeed, some type of mucous eruption in the part would probably be discerned as a common, if not constant phenomenon, if we had an opportunity of searching for it early in the course of the disease. Sometimes, but by no means frequently, there supervene one or two spots of ulceration, especially towards the orifice of the vagina.

*Treatment.*—Acute infantile leucorrœa shows, like many other analagous ailments, a natural tendency to run throug a definite, and often not a very long course, and has a natural tendency to end in a spontaneous cure. But if not arrested, it sometimes ends in a chronic, protracted form of discharge; and our medical interference is required, not only to prevent this termination, but also to relieve the patient from the immediate distress and annoyance which the disease produces.

In the first stages of the affection you will often find your patient requiring some little constitutional treatment. A dose of calomel, or grey powder, with a few grains of some aperient alkali, as magnesia, is sometimes all that is necessary; but it may require to be repeated. If the discharge becomes protracted, chalybeates or other tonics may become necessary. Use, in short, your constitutional remedies here as elsewhere, to bring the constitution and its principal functions as far as possible to the normal standard of health and cure the local disease by appropriate local applications.

In the earlier and more inflammatory periods of the disease, use locally to the vulva sedative applications; in the latter periods, and for the purpose of reducing and arresting the attendant discharge, use astringent applications. And you may employ these local applications in the form of lotions, or liniments, or ointments.

Frequent ablution with warm water, or warm milk and water, is one of the best and most soothing applications; and sitting in a warm cup-bath during micturition, is one of the surest means of relieving the pain and scalding attendant on the passage of the urine. A solution of acetate of lead and one grain of acetate of morphia to the ounce of water, makes a good sedative lotion where there is much local smarting and pain; or you may use a weak solution of boax, or of nitrate of silver. When sedative lotions are preferred, it is generally necessary to leave a slip of charpie, or lint, wetted with them, between the labia.

But I think you will find the local affection more manageable under sedative liniments; as by applying several times a day, with a brush or feather, cold cream, with, if you think fit, a little morphia added to it; or a liniment made of equal parts of olive oil and lime-water. Liniments are more lasting as local applications than lotions; and not so liable as ointments to fret or irritate.

But the time soon arrives when you must add local astringent remedies to the sedative, or substitute the former entirely for the latter. With this view, use tannin or sulphate of zinc, or aluminated iron, or any analogous astringent, in the form of a lotion or liniment; but take care not to use them of such strength that they prove irritant in their action, so as to force you back again, for a day or two, to the sedative treatment.

3. *Gangrenous Vulvitis or Norma.*—A variety of vulvitis seen sometimes, but fortunately very rarely, in practice, which generally begins with erythematous inflammation of the vulva, and the formation of one or two blisters or bullæ, and rapidly runs into one of gangrene and phagedæna. This gangrenous vulvitis has been seen in adults as a complication in particular epidemics of puerperal fever; and in infans it has been oberved either under an epidemic form, like epidemic gangrenous or malignant sore throat; or in isolated cases—most frequently as a sequela of scarlatina, measels, and other debilitating forms of ferbrile disease—or as a result of some constitutional cachexia. The disease not unfrequently runs on to a fatal termination.

*Treatment.*—You will generally find your patient requiring early, or even from the first, stimulants and quinine. If these are not yet called for, trust to considerable doses of chlorate of potass; or of this and muriate of iron, every two or three hours. Apply poultices and sedatives locally in the early stages of the disease, at least. The progress of the gangrene has, it is said, been sometimes arrested, or appeared to be arrested, by the application of nitric acid, &c.

4. *Prurigenous Vulvitis.*—Not unfrequently a prurigenous eruption appears on the mucous membrane of the vulva, and extends up along the vagina as far as the cervix uteri. It extends also often, and is sometimes, indeed, originally situated on the cutaneous border of the vulva, and appears on the outer cutaneoes surface of the labium, spreading backwards along the perineum to the circle of the anus. Occasionally, it is a flitting and transient affection, recurring with menstruation, pregnancy, or delivery. But patients will apply to you, from time to time, in whom the disease has become more chronic and fixed, having lasted for weeks, or months, or even years, producing almost constant irritation and distress; frequently interfering with rest and sleep, and rendering the victims of it miserable and almost deranged.—When the disease has become somewhat chronic, and necessitates the patient to attempt to alleviate it by constant and sometimes rough friction, you will find the mucous and even the cutaneous surface, at the most irritated parts, white and thickened with red fissures, and scratches appearing on the affected part. I have spoken of the disease as fundamentally prurigenous; and we can often see on the affected surface a small papular, and sometimes a vesicular, or even aphthous eruption; but cases ever and anon occur, of severe pruritus in these parts, without your being able to trace in them any distinct eruptive appearance.

*Treatment.*—Prurigenous disease of the vulva or neighborhood can be relieved, and generally cured, by the assiduous and persevering application of a solution of biborate of soda (five to ten grains to the ounce of water,) infusion of tobacco, either alone or containing a small quantity of borax dissolved in it, or an ointment of iodide of lead (one drachm to the ounce,) or an ointment of bismuth and morphi. Cloroform also applied locally, in the form of vapor, liniment, or ointment, forms one of the most certain means which you can use. The simplest way of employing it is, by adding a drachm of clorofarm to an ounce of any common or sedative ointment or linament. You will find great advantage in the management of the disease, in alternating some of these local applications with each other, for most of them begin to lose their good effects when persevered in above a few days consecutively. In the more obstinate and severe cases, strong astringents are sometimes of the greatest use, employed either alone or with the sedatives, as a very strong solution or ointment of alum or aluminated iron or tannin; or the powder of these substances mixed up with some powdered morphia, and applied continuously, for a few days, to the irritated part. Several times, in very obstinate cases, and where the disease was limited to a portion or circle of the cutaneous tissues, I have temporarily separated the affected portion of skin by a free subcutaneous incision, with a tenotomy knife. Of course, it leaves no wound, except by the entrance of the knife itself. I have found this operntion perfectly and entirely successful in some cases, and only of temporary benefit in others.

Perhaps it is unnecessary to add that the general health of the patient must be fully attended to, and that sometimes arsenic, aqua potassæ, and other alternative medicines of that description, are required.—*Medical Times and Gazette*, April 16, 1859.

